# Metabolic response during neuromuscular electrical stimulation in critically ill patients with deep sedation: a prospective observational study

**DOI:** 10.1186/s40635-026-01000-5

**Published:** 2026-11-10

**Authors:** Takuya Saeki, Masashi Yokose, Yusuke Saigusa, Miyu Kobayashi, Takeshi Nakamura

**Affiliations:** 1 Department of Rehabilitation Medicine, Yokohama City University Graduate School of Medicine, Yokohama, Japan; 2 Department of Rehabilitation Medicine, Yokohama City University Medical Center, Yokohama, Japan; 3 Department of Anesthesiology and Critical Care Medicine, Yokohama City University Graduate School of Medicine, 3-9, Fukuura, Kanazawa-ku, Yokohama, Japan; 4 Department of Biostatistics, Yokohama City University School of Medicine, Yokohama, Japan

**Keywords:** Early rehabilitation, Energy expenditure, Indirect calorimetry, Intensive care unit, Mechanical ventilation, Neuromuscular electrical stimulation, Sedation, Skeletal muscle atrophy

## Abstract

**Background:**

Neuromuscular electrical stimulation (NMES) is a potential strategy to counteract skeletal muscle atrophy in critically ill patients unable to engage in early mobilization. However, it is difficult to confirm whether the exercise load applied by NMES is sufficient to elicit a physiological effect in patients under sedation or with impaired consciousness, as the appropriate stimulus intensity and exercise load of NMES are generally assessed using subjective responses. This study aimed to evaluate changes in energy expenditure during NMES in sedated, mechanically ventilated patients using indirect calorimetry.

**Methods:**

This single-center observational study included 22 mechanically ventilated patients under deep sedation in the intensive care unit (ICU). Energy expenditure was measured using indirect calorimetry during NMES sessions. The primary outcome was the difference in energy expenditure before and during NMES. Multivariable linear regression analysis was performed to calculate the adjusted mean difference in energy expenditure. A clinically meaningful increase in energy expenditure attributable to NMES was predefined as 150 kcal/day, which was assumed based on an increase in energy expenditure when sitting on the edge of the bed.

**Results:**

In total, 26 NMES sessions involving 22 participants were included in the analysis. The mean energy expenditure was 1,137 ± 170 kcal/day pre-NMES and 1,350 ± 228 kcal/day during NMES. The adjusted increase in energy expenditure during NMES was 213.8 kcal/day (95% confidence interval, 137.1–290.3). This increase reached statistical significance when tested against the predefined threshold of 150 kcal/day (*P* = 0.02).

**Conclusions:**

The exercise load provided by NMES could be objectively assessed by measuring increases in energy expenditure. Although a statistically significant increase in energy expenditure was observed during NMES compared to pre-NMES levels, the magnitude of this increase was minimal. Further research is needed to determine whether NMES protocols that enhance energy expenditure are associated with muscle mass preservation, strength maintenance, and the prevention of ICU-acquired weakness.

**Supplementary Information:**

The online version contains supplementary material available at https://doi.org/10.1186/s40635-026-01000-5.

## Background

Among critically ill patients, advances in intensive care have substantially improved survival rates. However, significant concerns remain regarding functional outcomes, activities of daily living, and quality of life following discharge from the intensive care unit (ICU) [1]. Post-intensive care syndrome (PICS), characterized by complex impairments in physical and mental functions after ICU treatment, can persist long after hospital discharge [2]. ICU-acquired weakness (ICU-AW), a key component of PICS, manifests as rapid-onset, diffuse muscle weakness in the extremities of critically ill patients and is related to prolonged mechanical ventilation and extended ICU stays [3, 4]. Early rehabilitation therapy, particularly exercise-based interventions including mobilization within the first 48 h of ICU admission, is recommended to mitigate PICS [5, 6]. However, rehabilitation is often limited by respiratory or cardiovascular instability, the use of sedatives, and medical device-imposed constraints [7, 8].

In recent years, neuromuscular electrical stimulation (NMES) therapy has emerged as an alternative for maintaining skeletal muscle mass in critically ill patients unable to participate in early mobilization. NMES may help preserve muscle mass and potentially prevent ICU-AW [9–12], although some studies have reported no significant benefit compared with conventional rehabilitation [13–15]. The stimulus intensity of NMES is typically set based on a patient’s subjective assessment of their level of tolerable stimulation. However, in sedated or unconscious patients, evaluating subjective responses is challenging. For patients in the ICU, fluctuations in vital signs—including heart rate (HR) and blood pressure—resulting from autonomic nervous system responses are modified by factors such as a patient’s general physical condition and the effects of vasoactive agents, sedatives, and analgesics [16]. Currently, no objective indicator exists to determine whether NMES delivers a sufficient exercise load to induce physiological adaptations in skeletal muscle for sedated or unconscious patients, which may contribute to variability in NMES efficacy.

Indirect calorimetry provides estimates of metabolic parameters such as energy expenditure, volume of oxygen consumption (VO₂), and carbon dioxide production (VCO₂). In critically ill, mechanically ventilated patients, energy expenditure increases with activity intensity, such as sitting or standing during rehabilitation [17]. In healthy adults with obesity undergoing NMES, energy expenditure rises in a stimulus intensity–dependent manner [18]. These metabolic parameters may serve as objective indicators to optimize NMES stimulation intensity or duration in sedated patients or those with altered consciousness. However, it remains unclear whether critically ill patients with systemic inflammation and pharmacologic interventions respond to NMES in a manner comparable to healthy individuals.

This study aimed to evaluate changes in energy expenditure and respiratory metabolic response during NMES in sedated, mechanically ventilated patients by using indirect calorimetry.

## Methods

This prospective observational study was conducted in accordance with the Declaration of Helsinki and approved by the Institutional Review Board of Yokohama City University Hospital (approval no. B200600073; August 7, 2020). Written informed consent was obtained from all patients prior to inclusion. Patients admitted to the ICU of Yokohama City University Hospital between September 2020 and February 2022 were eligible if they: (i) met the inclusion criteria for early ICU rehabilitation (e.g., respiratory rate, < 35; oxygen saturation [SpO2] > 90%; fraction of inspired oxygen [FiO_2_], <​ 60%; no new episodes of severe arrhythmia or myocardial ischemia; mean arterial pressure, > 65 mmHg; HR, > 50 or < 120 beats per min; and no tendency to bleed) [19]; (ii) required mechanical ventilation with an expected duration of at least 24 h; (3) aged ≥ 20 years; and (4) were under sedation with a Richmond agitation-sedation scale (RASS) score of − 4 to − 5 attributable to therapeutic sedation or analgesia.

To minimize fluctuations in consciousness level and metabolic response variations as a result of sympathetic activation from NMES-related pain, only patients with a RASS score of − 4 to − 5, indicative of deep sedation, were included. Patients were excluded if they had implanted medical devices (e.g., pacemakers), prior thigh or lower leg amputation history, pregnancy or childbearing potential, disability from stroke or other central nervous system diseases, ventilator dependence with FiO_2_ > 60%, a respiratory rate >​ 35 breaths/min, respiratory leaks (e.g., pneumothorax), or a history of chronic obstructive pulmonary disease.

### NMES

NMES treatment sessions were conducted using a belt-type skeletal muscle electrical stimulator (G-TES^®^, Hormaion, Tokyo, Japan). Electrical stimulation was applied for 20 min at a frequency of 20 Hz, with a stimulation duration of 5 s and a pause duration of 3 s, aiming to enhance muscle strength. Stimulus intensity was determined using the quadriceps and triceps femoris muscles, as these provide stable contraction assessment in the supine position. Intensity was evaluated based on (i) visually confirmed contraction, (ii) palpable contraction, and (iii) no further changes in contraction magnitude or muscle stiffness despite increased current intensity [11, 13, 20]. Two medical staff assessed whether these criteria were met.

### Indirect calorimetry

VO₂, VCO₂, and end-tidal carbon dioxide (EtCO₂) were measured using an Engström Carestation (GE Healthcare, Chicago, IL, USA) system equipped with the modular metabolic monitor E-COVX™, specifically designed for mechanically ventilated patients. The measurement process for gas exchange has previously been described [21]. This system includes a pneumotachograph to measure inspired and expired volumes, a fast differential paramagnetic O₂ analyzer, and an infrared CO₂ analyzer. The pneumotachograph and gas sampling ports are housed in a disposable connector positioned between the endotracheal tube and the Y-piece of the ventilator circuit. Signals from the gas analyzers and the pneumotachograph are synchronized to provide breath-by-breath estimations of VO₂ and VCO₂.

### ICU management

All patients were sedated with propofol, fentanyl, and dexmedetomidine, titrated to maintain a RASS score of − 4 or − 5. Patients were ventilated using the Engström Carestation, targeting an end-tidal CO_2_ of 35–45 mmHg. The ventilator mode was selected at the discretion of the attending physician and remained unchanged during data collection. The FiO₂ setting was adjusted to maintain a partial pressure of oxygen level > 100 mmHg, as determined by arterial blood gas analysis. Vasopressors or vasodilators were adjusted at the physician’s discretion to maintain individualized blood pressure targets. The infusion rate for parenteral or enteral nutrition remained unchanged during data measurement.

### Data acquisition

Energy expenditure, VO_2_, VCO_2_, respiratory quotient (RQ), and vital signs were measured during three distinct periods: (i) for 10 min before NMES while the participant was at rest; (ii) during the NMES session (i.e., 20 min); and (iii) for 10 min after NMES, representing the recovery period. Arterial blood pressure (systolic [SAP], mean [MAP], and diastolic [DAP]), SpO₂, and HR were recorded using a bedside monitor (IntelliVue^®^ MX800, Philips Japan, Tokyo, Japan). Body temperature was measured via bladder temperature monitoring using an indwelling urinary catheter (Bardex Lubri-Sil I.C. Foley catheter; BARD, Covington, GA, USA). Data from the ventilator (i.e., respiratory rate, tidal volume, minute volume [MV], and EtCO_2_), metabolic parameters (i.e., energy expenditure, VO_2_, VCO_2_, and RQ measured using E-COVX™), and vital signs were automatically logged every minute in the ICU patient information system (ACSYS-Ki, Philips Japan) via the bedside monitor.

### Outcomes

The primary outcome was the difference in energy expenditure between the resting state and during NMES sessions. Secondary outcomes included: (i) the absolute difference and percentage increase in VO_2_, VCO_2_, and energy expenditure relative to the resting state; (ii) the difference in energy expenditure between the resting state and post-NMES recovery phase; and (iii) trends in mean and individual HR, SAP, MAP, EtCO_2_, and MV values across each study phase.

### Sample size calculation

For patients in the ICU, it remains unclear how much of an increase in energy expenditure is necessary through early rehabilitation to maintain physical function including muscle mass and muscle strength. Previous studies have reported that passive cycling or chair transfers—activities equivalent to an ICU Mobility Scale ≤​ 2—do not significantly increase energy expenditure or oxygen consumption [22, 23]. Thus, we assumed that the maximum exercise load feasible for intubated patients with impaired consciousness or sedation was sitting on the edge of the bed (ICU Mobility Scale 3), which is commonly performed in early rehabilitation in our institution. Hence, we assumed that an increase in energy expenditure attributable to NMES would be clinically significant if it exceeded the change in energy expenditure from that sitting on the edge of the bed. Based on our institutional data from four sedated patients, the mean difference in energy expenditure between resting and NMES was calculated as 268.8 ± 162.8 kcal/day. In a previous study that included a large number of patients who were not under deep sedation, the reported increase in energy expenditure from passive and active sitting on the edge of the bed was 115 kcal/day [17]. A clinically meaningful increase in energy expenditure with NMES was assumed to be 150 kcal/day, approximately 20% greater than that of sitting on the edge of the bed. Using a paired *t*-test with a significance level of 5%, we calculated that 22 sessions would provide 90% power to detect a mean within-session difference (i.e., during NMES and at rest) in energy expenditure with NMES exceeding the prespecified clinically meaningful threshold of 150 kcal/day. Allowing for potential dropouts, the planned sample size was 26 sessions.

### Statistical analysis

Vital signs, ventilator-related data, and metabolic data recorded every minute were extracted from the ACSYS-Ki database for analysis. Participant characteristics, vital signs, and indirect calorimetry data were transcribed into an Excel (Microsoft Corporation, Redmond, WA, USA) spreadsheet. Data points with RQ values ≤ 0.6 or ≥ 1.3 were considered measurement errors and excluded from the analysis. Energy expenditure, VO₂, and VCO₂ before, during, and after NMES sessions were calculated by averaging values within each measurement period. For the primary outcome, a multivariable linear regression model was fitted using generalized estimating equations, with energy expenditure measured at rest and during NMES as the outcome variable. Body mass index (BMI), age, and body temperature were included as explanatory variables. Observations from different sessions obtained from the same individuals were treated as independent. As energy expenditure measured at rest and during NMES within the same session was assumed to be correlated, generalized estimating equations with an exchangeable correlation structure were employed to account for within-session error correlations. The regression coefficient for NMES represents the mean difference in energy expenditure compared to rest; its estimate and standard error were used in a *Z*-test to evaluate whether the difference surpassed 150 kcal/day. For secondary outcomes, the mean differences in VO₂ and VCO₂ across measurement periods were tested using analysis of covariance with repeated measures. Additionally, a post-hoc analysis was conducted using the peak values of energy expenditure measured during the 10-min periods before, during, and 10-min periods after NMES sessions as representative values. All data are reported as numbers (percentages), medians (interquartile ranges), and means ± standard deviations, as appropriate. Statistical significance was set a​t a *P*-value < 0.05 (two-sided). All analyses were conducted using R software (version 4.2.2; The R Foundation for Statistical Computing, Vienna, Austria).

## Results

**Fig. 1:**
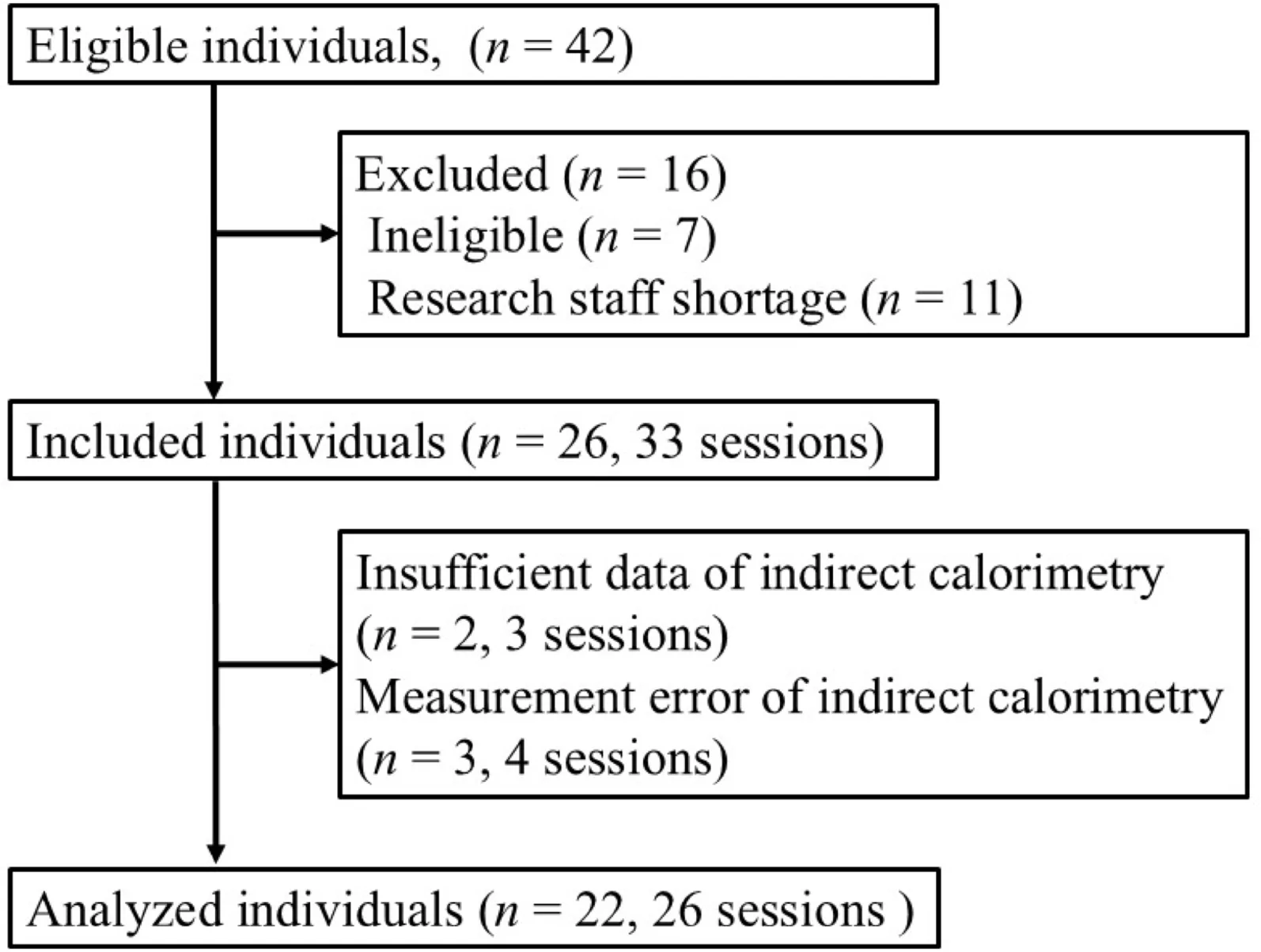
Study flowchart

Of the 33 NMES sessions conducted with 26 enrolled participants, seven sessions were excluded from the analysis. Three sessions were removed because of missing continuous data from indirect calorimetry, while four sessions were excluded owing to significant outliers in indirect calorimetry values, which were suspected to be measurement errors (Fig. 1). In the analysis, 26 NMES sessions from 22 participants were included. All participants were post-surgical patients in the ICU. In all included sessions, participants demonstrated an adequate physiological response to NMES stimulation based on a predefined standardized protocol. Every NMES session was completed as scheduled for the full 20 min. At all NMES sessions, no further adjustment to the ventilator’s FiO_2_ setting was required during indirect calorimetry measurements.

Table 1 presents the participants’ baseline characteristics. The median age was 70 (63–77) years, and 13 (59%) patients were men. The median BMI was 21.0 (19.0–22.2) kg/m². Table 2 presents clinical data at the time of NMES sessions. All participants received sedation with propofol, dexmedetomidine, and fentanyl. The number of NMES sessions conducted in deeply sedated patients (RASS, − 5) was 22 (85%) (Table 2).

**Table 1:** Participant characteristics

Characteristic	**All patients** ***(n*** **= 22)**
Age, years	70 (63–77)
Male sex, *n*	13 (59)
Height, cm	160 (155–167)
Weight, kg	55.4 (45.4–61.1)
BMI, kg/m^2^	21.0 (19.0–22.2)
ICU length of stay, days	4 (4 − 5)
Hospital length of stay, days	41 (26–47)
APACHE II score	20 (12–23)
SOFA score	7 (6–8)

All values were expressed as numbers (percentages) or medians (interquartile ranges)

APACHE, acute physiology and chronic health evaluation; BMI, body mass index; ICU, intensive care unit; SOFA, sequential organ failure assessment

**Table 2:** Clinical data at the time of NMES sessions

Variables	All sessions (*n* = 26 sessions)
Body temperature, ℃	37.4 (37.0–38.1)
Mechanical ventilation mode	
Pressure-controlled ventilation, *n*	19 (73)
Volume controlled ventilation, *n*	7 (27)
FiO_2_	0.40 (0.30–0.40)
Positive end-expiratory pressure, cmH_2_O	5 (5–5)
Respiratory rate, rate/min	12 (12–14)
Richmond agitation sedation scale, *n*	
−4	4 (15)
−5	22 (85)
Sedatives, *n*	
Propofol	26 (100)
Fentanyl	26 (100)
Dexmedetomidine	26 (100)
Midazolam	4 (15)
Vasopressor, *n*	20 (77)

All variables were expressed as numbers (percentages) or medians (interquartile ranges)

FiO_2_, fraction of inspiratory oxygen; *n*, number; NMES, neuromuscular electrical stimulation

### Primary outcome

**Fig. 2:**
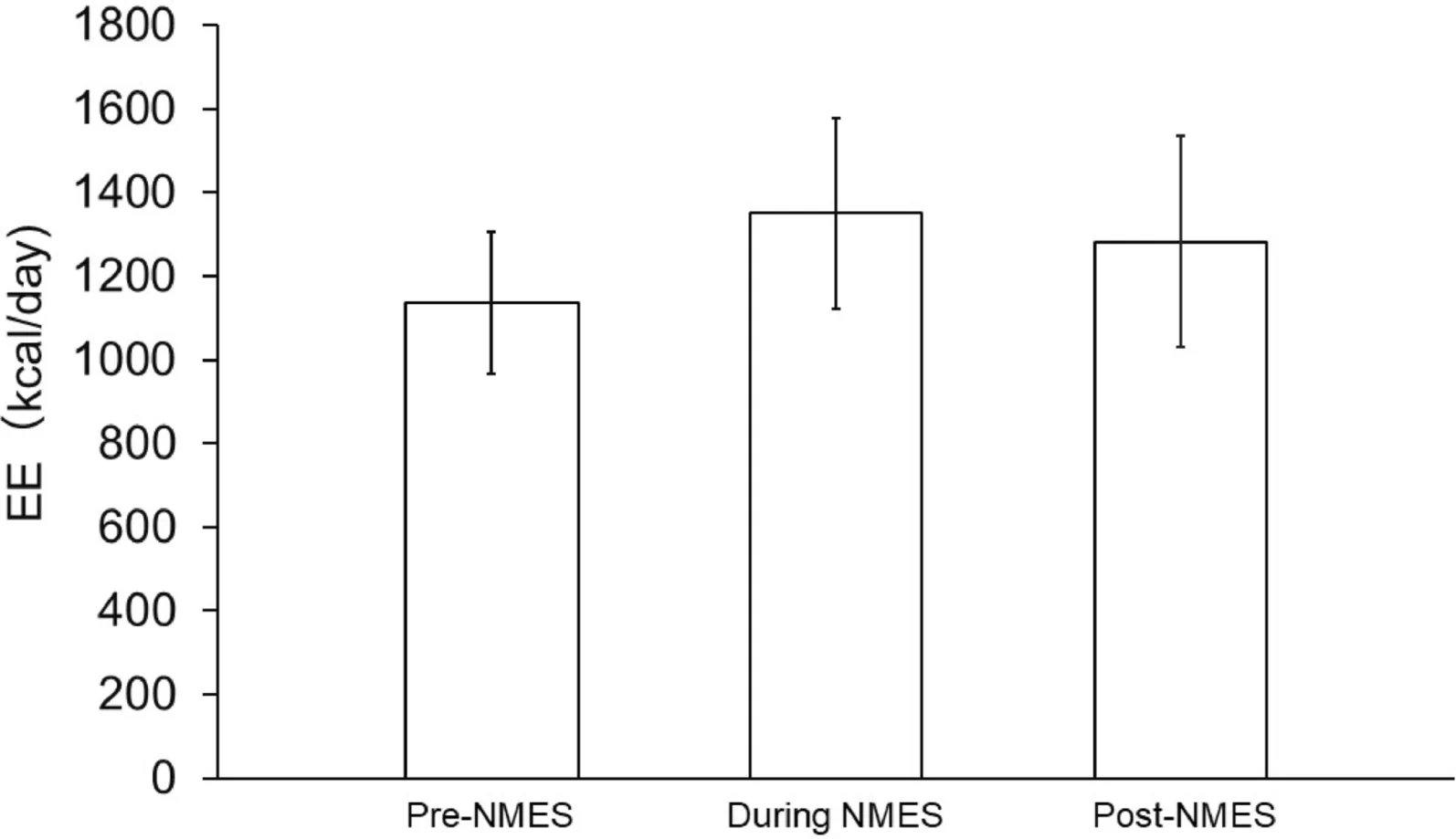
Energy expenditure across each phase. All data are presented as means, with error bars representing the standard deviation. Pre-NMES, 10-min measurement period before the NMES session; during-NMES, 20-min measurement period during NMES; post-NMES, 10-min measurement period following NMES. EE, energy expenditure; NMES, neuromuscular electrical stimulation

The energy expenditure for each study phase, as measured using indirect calorimetry, is shown in Fig. 2; Table 3. In Additional File 1, we present the trends in individual energy expenditure across each study phase. The mean energy expenditure (kcal/day) was 1,137 ± 170 pre-NMES, 1,350 ± 228 during NMES, and 1,281 ± 252 post-NMES. The percentage increase in energy expenditure from pre-NMES to during NMES and from pre-NMES to post-NMES was 19% (12–26) and 10% (6–17), respectively (Table 3). In primary analysis, the increase in energy expenditure during NMES, adjusted for BMI, age, and body temperature, was 213.8 kcal/day (95% confidence interval [CI] 161.2–266.3) (Table 4). This increase was statistically significantly higher than the 150 kcal/day assumed to represent a clinically meaningful increase in energy expenditure (*P* = 0.02) (Table 4). Prespecified additional analysis showed that NMES was also associated with an increase in post-NMES energy expenditure, adjusted for BMI, age, and body temperature, compared to resting levels (144.4 kcal/day; 95% CI 78.6–210; Table 4).

**Table 3:** Metabolic and hemodynamic parameters at each period

Metabolic and hemodynamic parameters	Pre-NMES	NMES	Post-NMES	*P*-value*
Energy expenditure (mean), (kcal/day)	1137 ± 170	1350 ± 228	1,281 ± 252	NA
Rate of increase in energy expenditure, (%)	Reference	19 (12–26)	10 (6–17)	NA
O_2_ consumption (mean), (mL/min)	166 ± 25	199 ± 33	185 ± 36	< 0.001
O_2_ consumption per body weight (mean), (mL/kg/min)	3.15 ± 0.56	3.77 ± 0.67	3.40 ± 0.68	< 0.001
Rate of increase in O_2_ consumption, (%)	Reference	19 (13–26)	9 (4–17)	NA
CO_2_ output (mean), (mL/min)	141 ± 21	159 ± 29	162 ± 32	< 0.01
Rate of increase in CO_2_ output, (%)	Reference	13 (7–16)	12 (6–18)	NA
HR, (beats/min)	73 ± 14.1	75 ± 15.0	76 ± 15.6	NA
SAP, (mmHg)	120 ± 17.5	115 ± 15.6	119 ± 17.9	NA
MAP, (mmHg)	75 ± 11.2	71 ± 10.4	74 ± 12.1	NA
EtCO_2_, (mmHg)	37 ± 4.0	40 ± 5.0	40 ± 4.7	NA
MV, (L/min)	5.6 ± 0.7	5.9 ± 0.9	5.8 ± 0.9	NA

Pre-NMES, 10-min measurement period before the NMES session; NMES, 20-min measurement period during the NMES session; post-NMES, 10-min measurement period after the NMES session

*P-value, the analysis of covariance for secondary outcome, i.e., O_2_ consumption and CO_2_

All variables were expressed as means ± standard deviations and medians (interquartile ranges)

CO_2,_ carbon dioxide; EtCO_2_, end-tidal carbon dioxide; HR, heart rate; MAP, mean arterial pressure; MV, minute volume; NA, not applicable; NMES, neuromuscular electrical stimulation; O_2_, oxygen; SAP, systolic arterial pressure

**Table 4:** Regression analysis results for energy expenditure using generalized estimating equations

Variables	Regression coefficients (95% CI) (kcal/day)	*P*-value	*P*-value (compared with 150 kcal/day)*
**Pre-NMES vs. during NMES**			
BMI	45.3 (16.7–73.9)	< 0.01	
Age	-5.9 (-10.7–-1.1)	0.016	
Body temperature	118.8 (42.1–195.5)	< 0.01	
NMES (vs. pre-NMES) (primary outcome)	213.8 (161.2–266.3)	< 0.001	0.02
**Pre-NMES vs. post-NMES**			
BMI	40.1 (9.6–70.6)	0.001	
Age	-3.3 (-9.4–2.8)	0.29	
Body temperature	124.6 (47.8–201)	0.001	
Post-NMES (vs. pre-NMES)	144.4 (78.6–210)	< 0.001	

*P-value, test for the null hypothesis that the mean difference in energy expenditure between rest and during the NMES session equals 150 kcal/day

Pre-NMES, 10-min measurement period before the NMES session; NMES, 20-min measurement period during the NMES session; post*-*NMES, 10-min measurement period after the NMES session

BMI, body mass index; CI, confidence interval; NMES, neuromuscular electrical stimulation

In the post-hoc analysis using peak energy expenditure values, the increase in adjusted energy expenditure (kcal/day) from rest was 339.1 (95% CI 255.4–442.8) during NMES and 273.8 (95% CI 188.9–358.6) post-NMES, showing a trend similar to that observed for the primary outcome measure (Additional File 2).

### Respiratory, metabolic, and hemodynamic responses

**Fig. 3:**
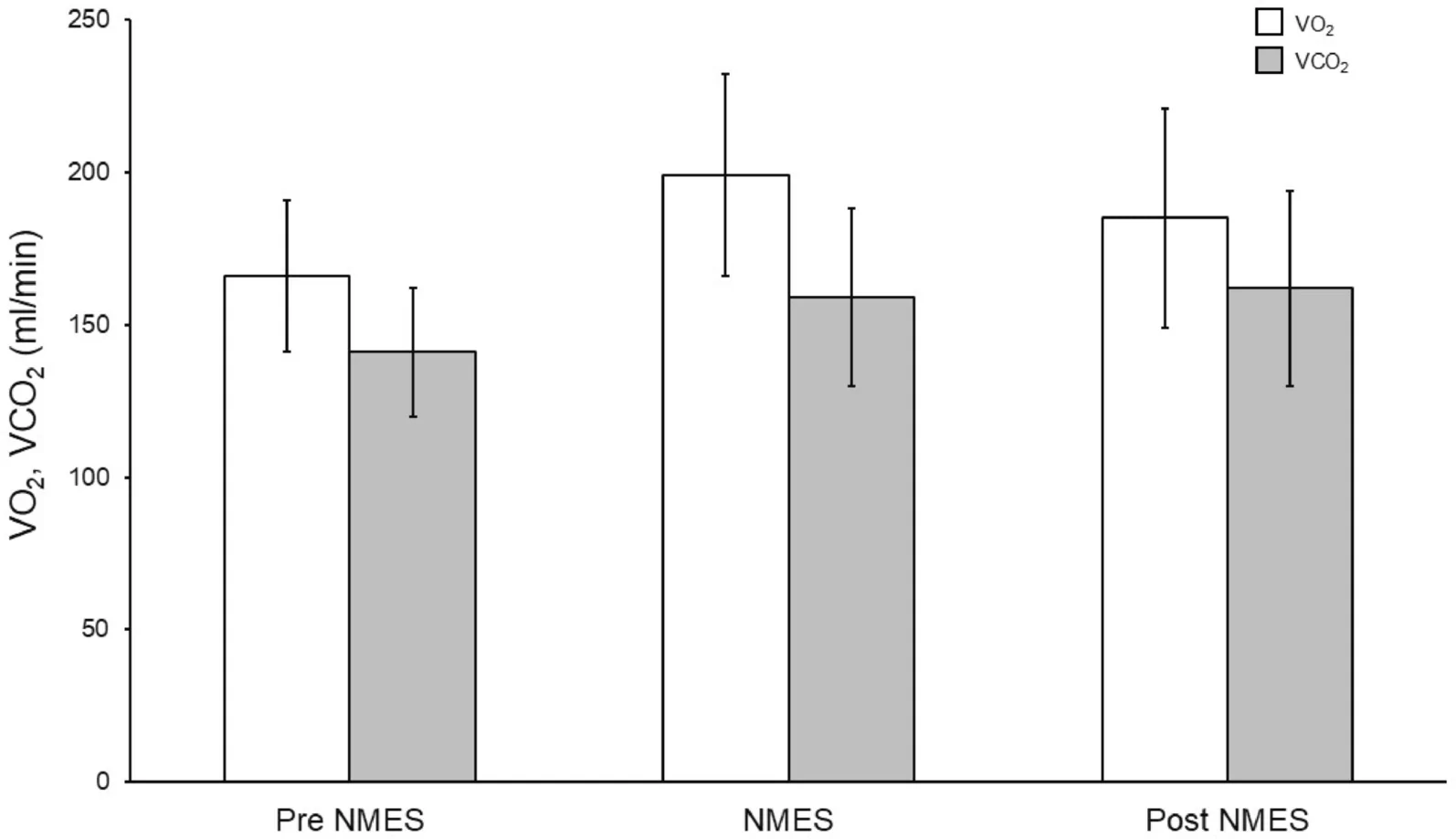
Oxygen consumption and carbon dioxide production across each study phase. White bar, VO₂; gray bar, VCO₂. All data are presented as means, with error bars representing the standard deviation. Pre-NMES, 10-min measurement period before the NMES session; during NMES, 20-min measurement period during NMES; post-NMES, 10-min measurement period following NMES. NMES, neuromuscular electrical stimulation; VO₂, oxygen consumption; VCO₂, carbon dioxide production

Figure 3; Table 3 show the VO₂ and VCO₂ values across all study phases. The mean VO₂ (mL/kg/min) was 3.15 ± 0.56 during the pre-NMES session, 3.77 ± 0.67 during NMES, and 3.50 ± 0.68 post-NMES. The percentage increase in VO₂ relative to pre-NMES values was 19% (13–26) when summarized using the mean VO₂. VCO₂ increased by 13% (7–16) above resting values when analyzed using the mean VCO₂ (Table 3). In the analysis of covariance adjusted for BMI, age, and body temperature, the mean differences in VO₂ and VCO₂ across measurement periods were statistically significant (Table 3).

Table 3 and Additional File 3 present the trends in mean and individual HR, SAP, MAP, EtCO_2_, and MV values across each study phase. The mean HR was 73 ± 14.1 beats/min at rest, 75 ± 15.0 during NMES, and 76 ± 15.6 post-NMES. SAP and MAP showed a slight decrease during NMES compared to baseline (SAP at rest: 120 ± 17.5 mmHg; SAP during NMES: 115 ± 15.6 mmHg; MAP at rest: 75 ± 11.2 mmHg; MAP during NMES: 71 ± 10.4 mmHg). EtCO_2_ and MV increased during and post-NMES compared to baseline (Table 3).

## Discussion

Our study findings indicated that energy expenditure, VO₂, and VCO₂ increased during NMES sessions in sedated critically ill patients, as measured using indirect calorimetry. For the primary outcome, energy expenditure during NMES increased by 213 kcal/day (i.e., 3.0 kcal/20 min) compared to pre-NMES levels, reflecting a 20% increase from baseline. This increase exceeded the increase in energy expenditure assumed to be clinically significant, as estimated based on sitting on the edge of the bed. The NMES-induced exercise load may be objectively evaluated through energy expenditure, even in the presence of systemic inflammation, sedatives, or catecholamines.

An increase in energy expenditure during NMES was observed compared to pre-NMES levels. VO₂ during NMES increased to 1.2 times the resting value, equivalent to the metabolic demand of standing quietly in a healthy adult (1.2 metabolic equivalents) [24]. Previous studies have shown that passive mobilization methods, such as passive cycling or chair transfers commonly used in sedated or unconscious patients, do not significantly increase energy expenditure or oxygen consumption [22, 23]. One study reported a mean difference in energy expenditure before and after passive cycling exercise of 84 kcal/day [22]. In a study primarily involving mechanically ventilated patients in the ICU who were not deeply sedated, energy expenditure increased by 0.4 kcal/5 min (i.e., 115 kcal/day) when sitting on the edge of the bed, 0.7 kcal/5 min (i.e., 221 kcal/day) for chair sitting, and 1.5 kcal/5 min (i.e., 473 kcal/day) for standing [17]. Our results showed that NMES may be a rehabilitation technique that increases energy expenditure to a degree comparable to sitting on the edge of the bed or transferring to a chair. However, the change in energy expenditure over the 20-minute NMES session was minimal (i.e., 3 kcal/20 min). If the goal is to improve exercise tolerance, or long-term functional independence, increasing the duration or frequency of NMES sessions or combining them with physical therapy and early mobilization may be necessary. The extent to which further increases in energy expenditure during NMES contribute clinically to maintaining physical function in critically ill patients remains unclear, and further research is needed.

The peak oxygen consumption of belt-type NMES has been reported to reach 3–4 metabolic equivalents in healthy adults [25] and significantly increase VO₂ compared to resting levels (3.2 ± 0.1 vs. 5.7 ± 0.1 mL/kg/min, respectively) [26]. However, in this study, NMES-induced increases in VO₂ (resting level: 3.2 ± 0.6 vs. NMES sessions: 3.8 ± 0.7 ml/kg/min); that is, the increase in energy expenditure was lower than that reported previously. Several factors may explain these differences. First, the exercise load applied using NMES may have been insufficient to elicit physiological effects on skeletal muscle mass and exercise tolerance. Second, ICU patient characteristics may have influenced the results. Critically ill patients with sepsis, edema, or vasopressor administration are less likely to achieve effective NMES-induced muscle contraction [16]. Muscle contraction efficiency is influenced by muscle mass and subcutaneous tissue thickness at the stimulation site [27, 28]. Our participants were older on average and included only post-surgical patients, such as those who had undergone free flap transfers for head and neck cancer reconstruction. Factors such as reduced muscle mass, postoperative edema, and inflammation may have affected NMES effectiveness [29, 30]. Finally, conducting this study in deeply sedated patients may have eliminated the increases in energy expenditure and VO₂ typically associated with pain or psychological stress during NMES. While the observed increases in energy expenditure and VO₂ were lower than those in previous studies, NMES remains one of the few interventions capable of providing an exercise load in patients who are sedated or unconscious. However, whether NMES protocols that increase energy expenditure and VO₂ are superior to those that do not remains unclear. Further research is needed to determine whether such increases translate into clinically meaningful benefits, and if so, the magnitude of increase required.

During the NMES sessions, energy expenditure remained elevated post-NMES compared to resting levels (Table 3). Excess post-exercise energy expenditure refers to the physiological response, in which VO₂ and energy expenditure remain above resting levels after exercise cessation [31]. The duration of this increase beyond the 10-min follow-up period in this study remains unclear. Evaluating energy expenditure and VO₂ not only during NMES, but also post-exercise may provide further insight into NMES physiological effects and its potential role in neuromuscular recovery and metabolic adaptation.

A NMES-related increase in energy expenditure was observed even in the presence of systemic inflammation, sedatives, and catecholamines. Currently, no standardized protocols define the purpose, indications, or methods of NMES, including parameters such as pulse duration, frequency, amplitude, duty cycle, duration, and intervention timing. Amplitude is typically set to “the maximum stimulus intensity that a patient can tolerate” [9, 10, 32]. Variability in NMES efficacy may stem from insufficient intensity caused by relying solely on visual contraction [33]. Establishing an objective index to evaluate NMES intensity is essential to determine whether the applied load is sufficient to elicit a hemodynamic response. The respiratory rate, arterial blood pressure, and HR typically respond to increased oxygen demand and can serve as objective indicators when subjective assessment by a patient is not feasible, as in critically ill patients. However, hemodynamic responses of critically ill patients may be influenced by sedative drugs, circulatory agonists, or systemic inflammation. In our study, HR and blood pressure showed minimal changes with NMES compared to the resting state (Table 3). Some individuals exhibited paradoxical responses, with HR and blood pressure decreasing after NMES initiation (Additional Files 3, 4). Therefore, indirect calorimetry may provide a more reliable method for quantifying NMES-induced exercise load across variations in pulse duration, frequency, amplitude, duty cycle, or duration. The subjective nature of intervention intensity measurement made standardization challenging and the interventions were not equal across our study patients; however, this approach could contribute to the development of standardized NMES protocols. To determine whether increased energy expenditure can serve as an indicator of high stimulation intensity and contraction quality, assessing changes in energy expenditure at different NMES stimulation intensities is essential in future studies.

### Limitations

Our study had some limitations. First, most study patients were postoperative patients who had undergone major head and neck cancer surgery with free flap reconstruction. Patients with lighter sedation, different medical conditions, or emergency admissions were not included, making it unclear whether similar results would be observed in these populations. Second, this was a small-sized, single-center observational study involving a subset of critically ill patients and may not be generalizable. Third, indirect calorimeters are not universally available across institutions, which may limit the generalizability of our findings. The precision of the E-COVX module is reported to be within 10% under FiO_2_ ≤ 0.40 conditions [34]. Therefore, when interpreting energy expenditure during NMES at an individual‑patient level, the potential influence of device‑related random measurement error should be considered, and cautious interpretation is warranted. Finally, we did not account for diet-induced thermogenesis, as nutritional therapy was not standardized; however, most eligible patients belonged to the same disease group, and data were collected during a period when continuous parenteral nutrition, rather than intermittent enteral nutrition, was administered.

## Conclusions

Energy expenditure measured using indirect calorimetry increased during NMES sessions in sedated, critically ill patients who were influenced by systemic inflammation, sedatives, or catecholamines increased compared to pre-NMES levels. This increase exceeded the increase in energy expenditure assumed to be clinically significant, as estimated based on sitting on the edge of the bed, but the magnitude of the increase was minimal. This objective approach using indirect calorimetry may aid in the development of optimized NMES protocols. Further research is needed to determine whether NMES protocols that enhance energy expenditure are associated with muscle mass preservation, strength maintenance, and the prevention of ICU-AW.

## Supplementary Information

Below is the link to the electronic supplementary material.

Supplementary Material 1

Supplementary Material 2

Supplementary Material 3

Supplementary Material 4

## Data Availability

The datasets used and/or analyzed during this study are available from the corresponding author on reasonable request.

## Abbreviations

APACHE: Acute physiology and chronic health evaluation
BMI: Body mass index
CI: Confidence interval
EtCO_2_: End-tidal carbon dioxide
FiO₂: Fraction of inspired oxygen
HR: Heart rate
ICU: Intensive care unit
ICU-AW: Intensive care unit-acquired weakness
MAP: Mean arterial pressure
MV: Minute volume
NMES: Neuromuscular electrical stimulation
PICS: Post-intensive care syndrome
RASS: Richmond agitation-sedation scale
RQ: Respiratory quotient
SAP: Systolic arterial pressure
SOFA: Sequential organ failure assessment
VCO_2_: Volume of carbon dioxide production
VO_2_: Volume of oxygen consumption
