# Supplementary material for: Metabolic response during neuromuscular electrical stimulation in critically ill patients with deep sedation: a prospective observational study
Source: Intensive Care Med Exp. 2026 Nov 10;14:145. doi: 10.1186/s40635-026-01000-5 (PMC13748173; doi:10.1186/s40635-026-01000-5)
Supplement: Supplementary file 1 [file 40635_2026_1000_MOESM1_ESM.docx]

**Additional File 1.** Trends in an individual’s energy expenditure parameters in an individual session

Energy expenditure was recorded breath‑by‑breath and averaged over 1‑min intervals for plotting. Measurements were performed during a 40‑min protocol consisting of rest (10 min), during NMES (20 min), and recovery (10 min). Each line represents an individual participant (a–z).

Abbreviations: NMES, neuromuscular electrical stimulation; min, minute
