# Supplementary material for: Metabolic response during neuromuscular electrical stimulation in critically ill patients with deep sedation: a prospective observational study
Source: Intensive Care Med Exp. 2026 Nov 10;14:145. doi: 10.1186/s40635-026-01000-5 (PMC13748173; doi:10.1186/s40635-026-01000-5)
Supplement: Supplementary file 2 [file 40635_2026_1000_MOESM2_ESM.docx]

**Additional File 2.** Post-hoc multiple regression analysis for peak value of energy expenditure.

| Variables | Regression coefficients (95% CI)  (kcal/day) | *P*-value |
| --- | --- | --- |
| **Rest vs. NMES** | | |
| BMI (each 1-point increment) | 53.4 (18.9–87.8) | 0.002 |
| Age (each 1-year increment) | −7.1 (−13.7 to −0.43) | 0.04 |
| Body temperature (each 1-degree increment) | 120.6 (30.7–210.4) | 0.008 |
| NMES (vs. at pre NMES) | 339.1 (255.4–442.8) | <0.001 |
| **Pre NMES vs. post NMES** | | |
| BMI (each 1-point increment) | 51.8 (16.9–86.7) | 0.004 |
| Age (each 1-year increment) | −5.3 (−12.1–1.55) | 0.13 |
| Body temperature (each 1-degree increment) | 122.6 (35.8–209.4) | 0.006 |
| Post-NMES (vs. at rest) | 273.8 (188.9–358.6) | <0.001 |

*Pre NMES* was a 10-min measurement period before NMES session. *NMES* was a 20-min measurement period during the NMES session. *Post*-NMES was a 10-min measurement period after the NMES session. *BMI* body mass index, *CI* confidence interval, *NMES* neuromuscular electrical stimulation
