# Supplementary material for: Metabolic response during neuromuscular electrical stimulation in critically ill patients with deep sedation: a prospective observational study
Source: Intensive Care Med Exp. 2026 Nov 10;14:145. doi: 10.1186/s40635-026-01000-5 (PMC13748173; doi:10.1186/s40635-026-01000-5)
Supplement: Supplementary file 3 [file 40635_2026_1000_MOESM3_ESM.docx]

**Additional File 3.** Trends in an individual’s hemodynamic and respiratory parameters.

**Heart rate**

HR (beats/min)

| Time (min.) |  |  |  |  |  |  |  |  |  |  |  |  |  |  |  |  |  |  |  |  |
| --- | --- | --- | --- | --- | --- | --- | --- | --- | --- | --- | --- | --- | --- | --- | --- | --- | --- | --- | --- | --- |
| 1 | 76 | 72 | 57 | 66 | 80 | 102 | 99 | 85 | 76 | 84 | 73 | 72 | 65 | 69 | 92 | 52 | 67 | 72 | 56 | 62 |
| 2 | 76 | 72 | 57 | 66 | 81 | 102 | 92 | 102 | 77 | 84 | 72 | 71 | 65 | 69 | 93 | 52 | 68 | 73 | 56 | 62 |
| 3 | 76 | 72 | 57 | 66 | 80 | 103 | 85 | 94 | 77 | 84 | 72 | 71 | 64 | 69 | 94 | 52 | 68 | 71 | 56 | 62 |
| 4 | 76 | 72 | 57 | 66 | 81 | 103 | 94 | 91 | 76 | 85 | 73 | 71 | 64 | 69 | 94 | 52 | 69 | 71 | 55 | 62 |
| 5 | 76 | 73 | 57 | 66 | 79 | 105 | 89 | 92 | 76 | 83 | 72 | 72 | 64 | 69 | 95 | 52 | 68 | 71 | 56 | 62 |
| 6 | 76 | 72 | 56 | 66 | 78 | 104 | 88 | 84 | 77 | 83 | 72 | 71 | 65 | 70 | 96 | 52 | 67 | 72 | 56 | 62 |
| 7 | 76 | 73 | 57 | 66 | 74 | 105 | 91 | 88 | 78 | 84 | 71 | 72 | 64 | 69 | 96 | 52 | 67 | 70 | 56 | 62 |
| 8 | 76 | 72 | 56 | 66 | 77 | 102 | 83 | 102 | 77 | 85 | 72 | 72 | 64 | 68 | 96 | 52 | 67 | 69 | 56 | 62 |
| 9 | 76 | 74 | 57 | 66 | 78 | 104 | 85 | 93 | 76 | 82 | 72 | 71 | 64 | 69 | 96 | 51 | 67 | 69 | 56 | 61 |
| 10 | 76 | 73 | 56 | 67 | 78 | 103 | 84 | 101 | 77 | 84 | 72 | 72 | 65 | 68 | 97 | 51 | 67 | 69 | 56 | 62 |
| 11 | 76 | 73 | 57 | 67 | 77 | 103 | 89 | 87 | 75 | 83 | 71 | 72 | 64 | 69 | 96 | 51 | 67 | 67 | 56 | 61 |
| 12 | 76 | 72 | 57 | 66 | 77 | 103 | 107 | 93 | 77 | 85 | 71 | 71 | 64 | 69 | 97 | 51 | 69 | 67 | 56 | 62 |
| 13 | 79 | 73 | 57 | 69 | 76 | 104 | 85 | 100 | 79 | 86 | 73 | 72 | 65 | 69 | 96 | 51 | 67 | 68 | 56 | 62 |
| 14 | 77 | 74 | 55 | 69 | 76 | 102 | 117 | 90 | 79 | 87 | 73 | 72 | 65 | 69 | 96 | 51 | 69 | 69 | 57 | 62 |
| 15 | 76 | 73 | 57 | 69 | 77 | 102 | 88 | 88 | 79 | 88 | 73 | 72 | 66 | 71 | 96 | 51 | 67 | 67 | 57 | 62 |
| 16 | 76 | 72 | 57 | 69 | 77 | 103 | 95 | 91 | 80 | 89 | 72 | 72 | 66 | 71 | 95 | 51 | 67 | 66 | 56 | 62 |
| 17 | 76 | 73 | 57 | 69 | 76 | 102 | 88 | 93 | 81 | 90 | 72 | 72 | 66 | 70 | 96 | 51 | 67 | 68 | 57 | 62 |
| 18 | 76 | 73 | 57 | 68 | 78 | 102 | 93 | 95 | 81 | 90 | 73 | 73 | 66 | 71 | 95 | 51 | 69 | 67 | 56 | 62 |
| 19 | 76 | 74 | 57 | 70 | 74 | 105 | 106 | 95 | 82 | 90 | 72 | 72 | 66 | 70 | 96 | 51 | 69 | 66 | 57 | 62 |
| 20 | 76 | 73 | 57 | 67 | 78 | 105 | 91 | 99 | 82 | 90 | 73 | 72 | 66 | 72 | 97 | 51 | 69 | 67 | 56 | 62 |
| 21 | 76 | 74 | 57 | 68 | 78 | 105 | 107 | 83 | 82 | 90 | 73 | 72 | 66 | 72 | 99 | 51 | 69 | 69 | 56 | 62 |
| 22 | 76 | 74 | 57 | 68 | 77 | 104 | 96 | 88 | 83 | 88 | 73 | 73 | 66 | 71 | 98 | 51 | 69 | 69 | 56 | 62 |
| 23 | 75 | 73 | 57 | 68 | 76 | 106 | 97 | 104 | 83 | 91 | 74 | 73 | 67 | 71 | 99 | 51 | 69 | 68 | 56 | 62 |
| 24 | 75 | 75 | 58 | 69 | 77 | 106 | 98 | 101 | 83 | 90 | 74 | 73 | 67 | 71 | 101 | 51 | 70 | 68 | 56 | 62 |
| 25 | 76 | 76 | 58 | 68 | 77 | 106 | 94 | 107 | 85 | 90 | 74 | 73 | 68 | 69 | 101 | 51 | 70 | 69 | 56 | 62 |
| 26 | 76 | 75 | 58 | 68 | 77 | 105 | 100 | 103 | 86 | 91 | 75 | 72 | 68 | 71 | 101 | 51 | 70 | 69 | 56 | 62 |
| 27 | 76 | 75 | 58 | 68 | 76 | 103 | 94 | 97 | 86 | 91 | 76 | 72 | 69 | 71 | 101 | 51 | 71 | 69 | 56 | 62 |
| 28 | 76 | 75 | 58 | 68 | 78 | 104 | 94 | 107 | 86 | 91 | 75 | 73 |  | 72 | 102 | 51 | 71 | 68 | 56 | 63 |
| 29 | 77 | 75 | 58 | 68 | 78 | 105 | 99 | 101 | 88 | 90 | 75 | 73 | 71 | 72 | 102 | 51 | 71 | 69 | 56 | 62 |
| 30 | 77 | 74 | 58 | 67 | 76 | 104 | 99 | 96 | 88 | 90 | 76 | 73 | 72 | 73 | 103 | 51 | 72 | 69 | 56 | 62 |
| 31 | 77 | 75 | 58 | 66 | 76 | 104 | 105 | 95 | 87 | 89 | 77 | 73 | 72 | 73 | 103 | 50 | 71 | 68 | 56 | 62 |
| 32 | 77 | 76 | 59 | 65 | 76 | 104 | 100 | 102 | 85 | 88 | 76 | 73 | 71 | 73 | 102 | 50 | 71 | 67 | 56 | 61 |
| 33 | 77 | 76 | 59 | 65 | 75 | 104 | 101 | 97 | 85 | 87 | 76 | 73 | 71 | 71 | 101 | 50 | 70 | 66 | 56 | 61 |
| 34 | 77 | 76 | 59 | 65 | 76 | 104 | 78 | 102 | 85 | 86 | 76 | 73 | 71 | 73 | 101 | 50 | 70 | 65 | 55 | 61 |
| 35 | 78 | 77 | 59 | 66 | 75 | 104 | 90 | 92 | 84 | 86 | 76 | 73 | 72 | 71 | 100 | 50 | 71 | 65 | 55 | 61 |
| 36 | 78 | 76 | 58 | 66 | 76 | 103 | 90 | 94 | 84 | 86 | 76 | 73 | 72 | 72 | 98 | 50 | 71 | 65 | 55 | 61 |
| 37 | 78 | 77 | 58 | 66 | 77 | 102 | 97 | 103 | 84 | 87 | 76 | 73 | 72 | 72 | 96 | 50 | 71 | 65 | 55 | 61 |
| 38 | 78 | 76 | 58 | 66 | 76 | 99 | 93 | 100 | 85 | 86 | 76 | 73 | 73 | 71 | 98 | 50 | 71 | 65 | 55 | 61 |
| 39 | 78 | 78 | 59 | 66 | 78 | 101 | 91 | 95 | 83 | 85 | 76 | 74 | 73 | 72 | 94 | 50 | 71 | 65 | 55 | 61 |
| 40 | 78 | 76 | 58 | 67 | 75 | 101 | 90 | 90 | 82 | 86 | 77 | 74 | 74 | 73 | 95 | 50 | 72 | 66 | 55 | 61 |

The red lines indicate sessions where the direction of change in heart rate after starting the neuromuscular electrical stimulation (NMES) was inconsistent with the direction of change in energy expenditure (EE). The number of sessions where the direction of change was the same was 21 (80.1%).

**Systolic arterial pressure**

Systolic arterial pressure (mmHg)

| Time (min) |  |  |  |  |  |  |  |  |  |  |  |  |  |  |  |  |  |  |  |  |
| --- | --- | --- | --- | --- | --- | --- | --- | --- | --- | --- | --- | --- | --- | --- | --- | --- | --- | --- | --- | --- |
| 1 | 118 | 100 | 114 | 94 | 112 | 119 | 135 | 110 | 100 | 92 | 118 | 132 | 156 | 121 | 121 | 149 | 112 | 159 | 129 | 108 |
| 2 | 117 | 100 | 106 | 93 | 114 | 119 | 135 | 114 | 101 | 92 | 116 | 134 | 160 | 127 | 122 | 146 | 105 | 157 | 129 | 107 |
| 3 | 120 | 100 | 108 | 93 | 112 | 121 | 130 | 114 | 100 | 94 | 117 | 135 | 162 | 125 | 118 | 146 | 106 | 154 | 129 | 108 |
| 4 | 119 | 101 | 108 | 94 | 111 | 118 | 137 | 115 | 101 | 93 | 114 | 135 | 159 | 124 | 118 | 144 | 110 | 153 |  | 108 |
| 5 | 119 | 103 | 110 | 93 | 111 | 118 | 132 | 116 | 101 | 95 | 115 | 134 | 162 | 121 | 118 | 143 | 112 | 149 | 128 | 109 |
| 6 | 118 | 103 | 112 | 94 | 110 | 117 | 134 | 112 | 103 | 94 | 113 | 135 | 159 | 120 | 126 | 143 | 113 | 145 | 130 | 116 |
| 7 | 120 | 104 | 109 | 93 | 105 | 118 | 140 | 115 | 104 | 95 | 115 | 135 | 157 | 121 | 124 | 144 | 112 | 141 | 128 | 105 |
| 8 | 121 | 105 | 108 | 93 | 108 | 121 | 136 | 114 | 105 | 97 | 116 | 138 | 160 | 119 | 137 | 143 | 111 | 138 | 129 | 104 |
| 9 | 120 | 107 | 109 | 94 | 110 | 122 | 137 | 117 | 105 | 94 | 115 | 138 | 158 | 119 | 130 | 141 | 112 | 137 | 128 | 103 |
| 10 | 121 | 106 | 107 | 93 | 110 | 121 | 140 | 116 | 105 | 95 | 115 | 141 | 156 | 117 | 129 | 141 | 111 | 136 | 134 | 104 |
| 11 | 121 | 105 | 109 | 93 | 111 | 120 | 138 | 111 | 104 | 97 | 113 | 140 | 154 | 120 | 142 | 140 | 112 | 133 | 128 | 102 |
| 12 | 120 | 105 | 108 | 92 | 112 | 111 | 140 | 117 | 105 | 95 | 112 | 139 | 157 | 119 | 140 | 143 | 104 | 131 | 125 | 97 |
| 13 | 128 | 107 | 105 | 87 | 108 | 113 | 131 | 117 | 106 | 99 | 111 | 138 | 150 | 119 | 143 | 138 | 99 | 127 | 120 | 96 |
| 14 | 123 | 109 | 101 | 89 | 108 | 113 | 135 | 116 | 106 | 100 | 111 | 135 | 144 | 117 | 128 | 137 | 95 | 128 | 118 | 95 |
| 15 | 106 | 111 | 105 | 87 | 108 | 111 | 131 | 115 | 103 | 96 | 112 | 131 | 140 | 118 | 124 | 134 | 92 | 128 | 114 | 94 |
| 16 | 103 | 108 | 101 | 87 | 107 | 114 | 131 | 118 | 103 | 94 | 114 | 129 | 139 | 116 | 126 | 137 | 92 | 125 | 110 | 93 |
| 17 | 105 | 110 | 100 | 87 | 107 | 118 | 130 | 112 | 109 | 100 | 115 | 129 | 141 | 115 | 130 | 138 | 91 | 120 | 109 | 94 |
| 18 | 108 | 110 | 100 | 87 | 109 | 121 | 127 | 113 | 110 | 102 | 115 | 131 | 141 | 117 | 129 | 139 | 90 | 119 | 109 | 93 |
| 19 | 108 | 112 | 99 | 89 | 107 | 119 | 131 | 114 | 111 | 103 | 117 | 134 | 142 | 116 | 130 | 137 | 92 | 118 | 110 | 93 |
| 20 | 108 | 112 | 96 | 93 | 109 | 114 | 123 | 116 | 111 | 104 | 123 | 130 | 140 | 117 | 132 | 136 | 91 | 121 | 110 | 94 |
| 21 | 107 | 114 | 119 | 93 | 110 | 114 | 128 | 113 | 114 | 104 | 127 | 129 | 140 | 118 | 134 | 136 | 89 | 122 | 111 | 93 |
| 22 | 107 | 114 | 122 | 92 | 109 | 113 | 124 | 111 | 114 | 101 | 128 | 132 | 139 | 117 | 129 | 136 | 88 | 123 | 111 | 92 |
| 23 | 106 | 114 | 125 | 90 | 108 | 111 | 124 | 116 | 113 | 102 | 134 | 131 | 139 | 116 | 126 | 135 | 86 | 122 | 111 | 93 |
| 24 | 103 | 116 | 128 | 91 | 109 | 112 | 125 | 115 | 113 | 100 | 134 | 131 | 139 | 115 | 130 | 132 | 85 | 122 | 110 | 92 |
| 25 | 105 | 116 | 128 | 91 | 108 | 112 | 123 | 111 | 115 | 95 | 135 | 130 | 137 | 116 | 128 | 132 | 80 | 122 | 109 | 92 |
| 26 | 107 | 116 | 128 | 91 | 109 | 116 | 120 | 112 | 112 | 96 | 134 | 129 | 137 | 116 | 125 | 131 | 84 | 120 | 109 | 91 |
| 27 | 109 | 117 | 129 | 91 | 108 | 115 | 123 | 114 | 114 | 102 | 132 | 131 |  | 115 | 129 | 130 | 84 | 119 | 108 | 91 |
| 28 | 111 | 117 | 125 | 90 | 110 | 118 | 121 | 117 | 114 | 100 | 132 | 133 | 134 | 116 | 125 | 128 | 83 | 118 | 107 | 90 |
| 29 | 112 | 117 | 125 | 91 | 109 | 118 | 122 | 115 | 118 | 94 | 133 | 135 | 136 | 116 | 124 | 127 | 83 | 120 | 106 | 91 |
| 30 | 114 | 120 | 124 | 91 | 110 | 117 | 120 | 118 | 117 | 91 | 133 | 134 | 140 | 116 | 125 | 125 | 82 | 119 | 106 | 91 |
| 31 | 114 | 121 | 125 | 91 | 112 | 116 | 120 | 121 | 115 | 95 | 134 | 136 | 145 | 116 | 135 | 124 | 82 | 119 | 107 | 91 |
| 32 | 115 | 117 | 124 | 92 | 113 | 121 | 120 | 120 | 114 | 102 | 132 | 136 | 149 | 116 | 124 | 125 | 81 | 119 | 108 | 92 |
| 33 | 116 | 115 | 125 | 92 | 113 | 113 | 119 | 126 | 116 | 103 | 131 |  | 150 | 115 | 122 | 130 | 84 | 120 | 110 | 94 |
| 34 | 118 | 115 | 123 | 94 | 115 | 119 | 120 | 125 | 117 | 101 | 132 | 141 | 150 | 117 | 134 | 131 | 86 | 118 | 109 | 94 |
| 35 | 117 | 114 | 123 | 94 | 114 | 113 | 123 | 125 | 116 | 99 | 131 | 141 | 150 | 114 | 140 | 132 | 88 | 119 | 110 | 94 |
| 36 | 116 | 112 | 123 |  | 113 | 120 | 122 | 127 | 114 | 99 | 132 | 141 | 154 | 118 | 153 | 132 | 89 | 118 | 110 | 92 |
| 37 | 116 | 111 | 122 |  | 112 | 120 | 124 | 128 | 115 | 104 | 132 | 143 | 160 | 115 | 161 | 133 | 89 | 116 | 110 | 92 |
| 38 | 115 | 111 | 119 | 87 | 111 | 120 | 123 | 131 | 114 | 99 | 131 | 141 | 148 | 127 | 161 | 133 | 92 | 116 | 110 | 91 |
| 39 | 114 | 111 | 118 | 92 | 113 | 117 | 124 | 126 | 113 | 89 | 131 | 141 | 148 | 129 | 180 | 131 | 90 | 117 | 110 | 92 |
| 40 | 113 | 111 | 129 | 91 | 110 | 116 | 121 | 128 | 112 | 88 | 131 | 140 | 156 | 124 | 181 | 131 | 91 | 117 | 111 | 92 |

The red lines represent sessions where the direction of change in systolic arterial pressure after starting the NMES was inconsistent with the direction of change in EE. The number of sessions where the direction of change was the same was 7 (26.9%).

*NMES* neuromuscular electrical stimulation, *EE* energy expenditure

**Mean arterial pressure**

Mean arterial pressure ()mmHg

| Time (min) |  |  |  |  |  |  |  |  |  |  |  |  |  |  |  |  |  |  |  |  |
| --- | --- | --- | --- | --- | --- | --- | --- | --- | --- | --- | --- | --- | --- | --- | --- | --- | --- | --- | --- | --- |
| 1 | 80 | 63 | 62 | 66 | 65 | 73 | 98 | 75 | 64 | 58 | 76 | 68 | 83 | 77 | 90 | 87 | 79 | 96 | 81 | 71 |
| 2 | 79 | 62 | 58 | 65 | 67 | 72 | 98 | 78 | 64 | 58 | 74 | 68 | 84 | 79 | 91 | 84 | 76 | 95 | 80 | 70 |
| 3 | 81 | 63 | 58 | 65 | 65 | 74 | 93 | 79 | 64 | 59 | 75 | 69 | 85 | 78 | 89 | 85 | 78 | 94 | 81 | 71 |
| 4 | 80 | 63 | 58 | 66 | 65 | 72 | 98 | 80 | 64 | 58 | 73 | 69 | 83 | 78 | 89 | 84 | 79 | 95 |  | 71 |
| 5 | 80 | 64 | 59 | 66 | 64 | 73 | 94 | 81 | 65 | 59 | 74 | 68 | 84 | 77 | 89 | 83 | 80 | 92 | 80 | 72 |
| 6 | 79 | 64 | 61 | 66 | 65 | 72 | 95 | 78 | 65 | 59 | 73 | 68 | 82 | 77 | 94 | 83 | 81 | 90 | 81 | 75 |
| 7 | 81 | 65 | 58 | 65 | 62 | 73 | 101 | 80 | 66 | 60 | 75 | 70 | 81 | 77 | 91 | 84 | 80 | 88 | 81 | 67 |
| 8 | 81 | 65 | 58 | 65 | 63 | 74 | 97 | 80 | 66 | 60 | 75 | 70 | 83 | 75 | 101 | 83 | 79 | 86 | 81 | 66 |
| 9 | 81 | 66 | 58 | 66 | 65 | 74 | 100 | 83 | 67 | 59 | 75 | 71 | 82 | 76 | 97 | 81 | 79 | 85 | 81 | 65 |
| 10 | 81 | 65 | 56 | 65 | 65 | 74 | 100 | 81 | 67 | 60 | 75 | 72 | 80 | 74 | 95 | 81 | 79 | 84 | 84 | 66 |
| 11 | 81 | 65 | 58 | 65 | 66 | 73 | 99 | 78 | 66 | 62 | 74 | 71 | 79 | 76 | 104 | 81 | 80 | 83 | 81 | 65 |
| 12 | 80 | 65 | 57 | 64 | 66 | 68 | 99 | 81 | 67 | 59 | 74 | 71 | 81 | 76 | 102 | 83 | 75 | 81 | 79 | 60 |
| 13 | 84 | 66 | 56 | 60 | 63 | 70 | 93 | 81 | 68 | 61 | 73 | 70 | 78 | 76 | 104 | 78 | 71 | 78 | 75 | 59 |
| 14 | 81 | 66 | 53 | 61 | 63 | 69 | 97 | 81 | 66 | 63 | 73 | 68 | 74 | 73 | 94 | 77 | 69 | 80 | 74 | 59 |
| 15 | 68 | 67 | 55 | 60 | 63 | 67 | 93 | 80 | 65 | 62 | 74 | 66 | 73 | 74 | 92 | 75 | 67 | 80 | 71 | 59 |
| 16 | 67 | 66 | 53 | 59 | 62 | 69 | 94 | 81 | 65 | 62 | 75 | 66 | 72 | 72 | 93 | 77 | 67 | 76 | 69 | 57 |
| 17 | 68 | 66 | 52 | 59 | 62 | 71 | 92 | 78 | 67 | 62 | 76 | 66 | 74 | 72 | 95 | 79 | 66 | 74 | 69 | 58 |
| 18 | 70 | 66 | 52 | 59 | 63 | 73 | 90 | 78 | 67 | 63 | 76 | 67 | 74 | 73 | 95 | 79 | 65 | 74 | 68 | 56 |
| 19 | 70 | 67 | 51 | 61 | 62 | 72 | 93 | 79 | 68 | 63 | 77 | 68 | 75 | 72 | 95 | 77 | 66 | 74 | 68 | 57 |
| 20 | 70 | 67 | 50 | 65 | 64 | 70 | 88 | 80 | 68 | 64 | 81 | 66 | 73 | 73 | 96 | 76 | 66 | 76 | 68 | 57 |
| 21 | 69 | 68 | 68 | 64 | 64 | 69 | 90 | 79 | 68 | 64 | 85 | 65 | 73 | 72 | 97 | 76 | 65 | 76 | 69 | 57 |
| 22 | 69 | 67 | 69 | 63 | 63 | 69 | 88 | 77 | 68 | 61 | 90 | 66 | 73 | 72 | 95 | 75 | 64 | 76 | 69 | 56 |
| 23 | 67 | 68 | 71 | 62 | 62 | 67 | 88 | 80 | 67 | 63 | 89 | 66 | 73 | 71 | 93 | 74 | 62 | 75 | 68 | 56 |
| 24 | 68 | 68 | 72 | 62 | 63 | 69 | 90 | 79 | 68 | 63 | 90 | 66 | 73 | 70 | 95 | 73 | 63 | 76 | 68 | 55 |
| 25 | 70 | 68 | 72 | 62 | 63 | 69 | 87 | 78 | 68 | 65 | 90 | 65 | 73 | 71 | 94 | 72 | 59 | 74 | 67 | 56 |
| 26 | 71 | 68 | 72 | 62 | 63 | 71 | 85 | 78 | 67 | 65 | 90 | 65 | 73 | 71 | 92 | 72 | 59 | 74 | 67 | 55 |
| 27 | 72 | 68 | 72 | 62 | 62 | 70 | 88 | 75 | 68 | 64 | 89 | 65 |  | 70 | 95 | 71 | 59 | 73 | 67 | 55 |
| 28 | 73 | 68 | 71 | 61 | 64 | 72 | 86 | 79 | 68 | 62 | 90 | 66 | 73 | 70 | 91 | 70 | 59 | 73 | 66 | 54 |
| 29 | 74 | 68 | 71 | 62 | 63 | 72 | 88 | 82 | 69 | 64 | 90 | 67 | 75 | 70 | 89 | 69 | 58 | 73 | 66 | 55 |
| 30 | 75 | 69 | 70 | 62 | 63 | 72 | 84 | 80 | 69 | 65 | 91 | 67 | 78 | 71 | 92 | 68 | 58 | 73 | 66 | 55 |
| 31 | 75 | 69 | 71 | 62 | 65 | 72 | 85 | 82 | 68 | 64 | 91 | 68 | 79 | 71 | 99 | 68 | 58 | 74 | 67 | 55 |
| 32 | 76 | 67 | 71 | 64 | 66 | 75 | 86 | 83 | 68 | 64 | 91 | 68 | 81 | 71 | 90 | 69 | 60 | 76 | 68 | 57 |
| 33 | 78 | 67 | 72 | 64 | 67 | 71 | 86 | 85 | 69 | 64 | 90 |  | 83 | 71 | 89 | 73 | 59 | 77 | 69 | 58 |
| 34 | 77 | 67 | 70 | 66 | 68 | 70 | 86 | 88 | 70 | 64 | 90 | 70 | 82 | 72 | 101 | 74 | 60 | 77 | 69 | 58 |
| 35 | 76 | 66 | 71 | 66 | 67 | 71 | 87 | 86 | 69 | 63 | 90 | 70 | 82 | 71 | 104 | 75 | 62 | 77 | 70 | 58 |
| 36 | 77 | 66 | 70 |  | 67 | 75 | 87 | 87 | 69 | 63 | 91 | 70 | 83 | 73 | 112 | 74 | 63 | 76 | 70 | 57 |
| 37 | 76 | 65 | 70 |  | 66 | 74 | 87 | 88 | 69 | 65 | 91 | 72 | 87 | 72 | 115 | 75 | 63 | 76 | 70 | 57 |
| 38 | 75 | 65 | 68 | 63 | 66 | 75 | 88 | 89 | 69 | 63 | 89 | 71 | 81 | 75 | 116 | 75 | 65 | 76 | 69 | 57 |
| 39 | 74 | 65 | 68 | 66 | 67 | 73 | 90 | 91 | 68 | 64 | 89 | 72 | 81 | 78 | 128 | 74 | 64 | 76 | 70 | 57 |
| 40 |  | 65 | 74 | 65 | 65 | 72 | 86 | 87 | 67 | 69 | 88 | 72 | 84 | 77 | 129 | 73 | 64 | 77 | 70 | 57 |

The red lines indicate sessions where the direction of change in mean arterial pressure after starting the NMES was inconsistent with the direction of change in EE. The number of sessions where the direction of change was the same was 6 (23.1%).

*NMES* neuromuscular electrical stimulation, *EE* energy expenditure

**End tidal carbon dioxide**

EtCO2 (mmHg)

| Time (min) |  |  |  |  |  |  |  |  |  |  |  |  |  |  |  |  |  |  |  |  |
| --- | --- | --- | --- | --- | --- | --- | --- | --- | --- | --- | --- | --- | --- | --- | --- | --- | --- | --- | --- | --- |
| 1 | 30 | 34 | 38 | 40 | 38 | 43 | 36 | 34 | 38 | 39 | 45 | 40 | 37 | 40 | 36 | 37 | 40 | 37 | 32 | 32 |
| 2 | 30 | 35 | 38 | 40 | 38 | 42 | 36 | 34 | 38 | 41 | 47 | 40 | 38 | 41 | 36 | 37 | 40 | 37 | 32 | 32 |
| 3 | 31 | 34 | 39 | 40 | 38 | 42 | 35 | 34 | 38 | 41 | 46 | 40 | 37 | 41 | 37 | 37 | 40 | 36 | 32 | 33 |
| 4 | 31 | 35 | 38 | 40 | 38 | 41 | 36 | 34 | 38 | 40 | 46 | 41 | 37 | 41 | 36 | 37 | 40 | 37 | 32 | 32 |
| 5 | 31 | 35 | 38 | 39 | 37 | 41 | 35 | 34 | 38 | 41 | 47 | 40 | 37 | 41 | 36 | 37 | 40 | 36 | 32 | 33 |
| 6 | 31 | 35 | 39 | 39 | 38 | 41 | 36 | 34 | 38 | 40 | 47 | 40 | 37 | 41 | 36 | 37 | 41 | 36 | 32 | 32 |
| 7 | 31 | 34 | 38 | 39 | 37 | 41 | 36 | 34 | 38 | 41 | 46 | 40 | 37 | 40 | 36 | 37 | 40 | 36 | 32 | 32 |
| 8 | 30 | 34 | 38 | 39 | 37 | 41 | 36 | 34 | 38 | 40 | 45 | 40 | 37 | 41 | 36 | 37 | 40 | 36 | 32 | 32 |
| 9 | 31 | 35 | 38 | 39 | 37 | 41 | 36 | 34 | 38 | 39 | 44 | 41 | 37 | 40 | 36 | 37 | 39 | 36 | 32 | 32 |
| 10 | 31 | 35 | 39 | 39 | 36 | 41 | 36 | 34 | 38 | 40 | 44 | 40 | 37 | 40 | 36 | 37 | 40 | 36 | 32 | 32 |
| 11 | 31 | 35 | 39 | 39 | 37 | 41 | 36 | 34 | 38 | 41 | 43 | 40 | 37 | 40 | 36 | 37 | 40 | 36 | 32 | 32 |
| 12 | 31 | 35 | 39 | 39 | 36 | 42 | 35 | 34 | 38 | 41 | 43 | 40 | 36 | 40 | 36 | 37 | 40 | 36 | 32 | 31 |
| 13 | 32 | 34 | 39 | 40 | 36 | 42 | 36 | 34 | 39 | 40 | 43 | 40 | 36 | 41 | 36 | 37 | 42 | 36 | 33 | 34 |
| 14 | 33 | 34 | 40 | 41 | 37 | 43 | 37 | 34 | 40 | 40 | 42 | 41 | 39 | 42 | 39 | 37 | 43 | 36 | 33 | 34 |
| 15 | 33 | 34 | 40 | 43 | 38 | 43 | 37 | 36 | 44 | 42 | 41 | 42 | 40 | 42 | 42 | 39 | 44 | 36 | 35 | 36 |
| 16 | 33 | 34 | 40 | 45 | 39 | 43 | 37 | 36 | 44 | 40 | 41 | 43 | 41 | 43 | 43 | 38 | 45 | 38 | 36 | 36 |
| 17 | 34 | 34 | 40 | 45 | 39 | 43 | 37 | 36 | 44 | 41 | 41 | 44 | 43 | 43 | 46 | 39 | 45 | 38 | 37 | 38 |
| 18 | 35 | 34 | 40 | 44 | 39 | 43 | 38 | 36 | 44 | 40 | 41 | 45 | 43 | 44 | 46 | 40 | 45 | 39 | 38 | 38 |
| 19 | 36 | 34 | 41 | 41 | 39 | 41 | 38 | 37 | 44 | 40 | 40 | 46 | 43 | 45 | 48 | 39 | 46 | 38 | 38 | 38 |
| 20 | 36 | 34 | 41 | 44 | 39 | 42 | 38 | 37 | 45 | 41 | 40 | 47 | 43 | 45 | 49 | 40 | 46 | 37 | 38 | 38 |
| 21 | 36 | 34 | 42 | 45 | 40 | 40 | 39 | 38 | 48 | 41 | 41 | 47 | 44 | 45 | 49 | 40 | 48 | 37 | 38 | 38 |
| 22 | 36 | 34 | 41 | 44 | 41 | 40 | 39 | 36 | 46 | 42 | 40 | 47 | 45 | 45 | 49 | 40 | 47 | 38 | 38 | 38 |
| 23 | 36 | 34 | 42 | 44 | 41 | 40 | 39 | 35 | 48 | 42 | 40 | 47 | 45 | 45 | 49 | 40 | 48 | 39 | 38 | 39 |
| 24 | 36 | 34 | 42 | 46 | 41 | 40 | 40 | 37 | 47 | 41 | 41 | 47 | 43 | 45 | 48 | 40 | 48 | 38 | 38 | 39 |
| 25 | 36 | 34 | 42 | 44 | 41 | 41 | 39 | 36 | 50 | 41 | 41 | 47 | 41 | 46 | 49 | 40 | 49 | 38 | 38 | 39 |
| 26 | 36 | 34 | 42 | 44 | 42 | 42 | 39 | 35 | 49 | 43 | 41 | 47 | 44 | 45 | 48 | 40 | 49 | 39 | 38 | 39 |
| 27 | 36 | 34 | 42 | 44 | 41 | 41 | 40 | 36 | 48 | 41 | 41 | 48 |  | 46 | 49 | 41 | 49 | 39 | 38 | 39 |
| 28 | 37 | 34 | 42 | 43 | 42 | 41 | 38 | 36 | 51 | 42 | 41 | 47 | 43 | 45 | 48 | 40 | 49 | 39 | 37 | 40 |
| 29 | 37 | 34 | 42 | 44 | 41 | 41 | 38 | 34 | 51 | 41 | 41 | 48 | 43 | 45 | 48 | 41 | 49 | 39 | 37 | 40 |
| 30 | 37 | 34 | 41 | 43 | 41 | 41 | 38 | 36 | 51 | 42 | 41 | 48 | 43 | 45 | 46 | 42 | 49 | 39 | 37 | 39 |
| 31 | 37 | 34 | 40 | 42 | 41 | 41 | 38 | 36 | 50 | 42 | 41 | 48 | 43 | 46 | 47 | 42 | 48 | 39 | 37 | 39 |
| 32 | 36 | 34 | 40 | 42 | 40 | 41 | 37 | 36 | 48 | 41 | 41 | 47 | 43 | 45 | 46 | 41 | 49 | 36 | 36 | 38 |
| 33 | 36 | 34 | 40 | 42 | 40 | 40 | 38 | 34 | 47 | 40 | 41 | 47 | 43 | 45 | 45 | 41 | 47 | 36 | 35 | 38 |
| 34 | 35 | 34 | 40 | 41 | 39 | 40 | 37 | 34 | 49 | 39 | 41 | 45 | 43 | 44 | 43 | 40 | 46 | 36 | 35 | 37 |
| 35 | 35 | 34 | 39 | 41 | 39 | 40 | 37 | 34 | 45 | 41 | 41 | 46 | 46 | 44 | 41 | 40 | 46 | 36 | 35 | 36 |
| 36 | 34 | 34 | 39 | 41 | 39 | 40 | 37 | 34 | 44 | 42 | 42 | 44 | 43 | 44 | 43 | 40 | 45 | 35 | 35 | 36 |
| 37 | 34 | 34 | 39 | 41 | 39 | 40 | 37 | 34 | 43 | 42 | 41 | 44 | 43 | 43 | 41 | 40 | 45 | 34 | 35 | 35 |
| 38 | 33 | 34 | 39 | 41 | 39 | 40 | 37 | 34 | 43 | 42 | 41 | 44 | 44 | 43 | 42 | 39 | 45 | 34 | 34 | 35 |
| 39 | 34 | 34 | 37 | 40 | 38 | 40 | 37 | 34 | 44 | 40 | 41 | 44 | 43 | 43 | 39 | 39 | 44 | 35 | 34 | 34 |
| 40 | 33 | 34 | 39 | 40 | 37 | 40 | 36 | 34 | 43 | 42 | 41 | 44 | 44 | 43 | 40 | 39 | 44 | 35 | 33 | 34 |

The red lines show sessions where the direction of change in end tidal carbon dioxide after starting the NMES was inconsistent with the direction of change in EE. The number of sessions where the direction of change was the same was 24 (92.3%).

*NMES* neuromuscular electrical stimulation, *EE* energy expenditure

**Minute volume**

The red lines represent sessions where the direction of change in minute volume after starting the NMES was inconsistent with the direction of change in EE. The number of sessions where the direction of change was the same was 19 (73.1%).

Minute Volume (L/min)

| Time (min) |  |  |  |  |  |  |  |  |  |  |  |  |  |  |  |  |  |  |  |  |
| --- | --- | --- | --- | --- | --- | --- | --- | --- | --- | --- | --- | --- | --- | --- | --- | --- | --- | --- | --- | --- |
| 1 | 6.5 | 5.6 | 4.5 | 5.6 | 5.8 | 4.9 | 5.8 | 5.8 | 6 | 6.6 | 5.7 | 5.7 | 5.7 | 4.8 | 5.6 | 5.4 | 4.1 | 6.1 | 6.3 | 4.9 |
| 2 | 6.5 | 5.6 | 4.5 | 5.5 | 5.7 | 4.9 | 5.8 | 5.8 | 6 | 6.5 | 5.6 | 5.7 | 5.7 | 5 | 5.6 | 5.4 | 4.1 | 6.1 | 6.4 | 4.8 |
| 3 | 6.5 | 5.6 | 4.5 | 5.4 | 5.7 | 4.9 | 5.9 | 5.9 | 6 | 6.6 | 5.5 | 5.7 | 5.7 | 4.8 | 5.5 | 5.3 | 4.2 | 6.2 | 6.3 | 4.9 |
| 4 | 6.5 | 5.6 | 4.5 | 5.6 | 5.7 | 4.9 | 5.9 | 5.9 | 6 | 6.1 | 5.7 | 5.7 | 5.8 | 4.8 | 5.6 | 5.2 | 4.2 | 6.1 | 6.3 | 4.9 |
| 5 | 6.5 | 5.6 | 4.6 | 5.5 | 5.6 | 4.9 | 5.9 | 5.8 | 6 | 6.3 | 5.7 | 5.7 | 5.7 | 4.8 | 5.6 | 5.2 | 4.1 | 5.7 | 6.3 | 4.9 |
| 6 | 6.5 | 5.6 | 4.7 | 5.5 | 5.7 | 4.9 | 5.8 | 5.9 | 6 | 6.4 | 5.7 | 5.7 | 5.7 | 4.8 | 5.6 | 5.2 | 4.1 | 5.6 | 6.5 | 4.8 |
| 7 | 6.5 | 5.6 | 4.5 | 5.4 | 5.7 | 4.9 | 5.9 | 5.8 | 6 | 6.4 | 6.1 | 5.7 | 5.7 | 4.8 | 5.6 | 5.2 | 4.1 | 5.6 | 6.5 | 4.8 |
| 8 | 6.5 | 5.5 | 4.5 | 5.5 | 5.7 | 4.9 | 5.8 | 5.8 | 6 | 6.4 | 5.8 | 5.7 | 5.7 | 4.8 | 5.6 | 5.2 | 4.1 | 5.6 | 6.2 | 4.9 |
| 9 | 6.4 | 5.6 | 4.5 | 5.5 | 5.7 | 4.9 | 5.9 | 5.9 | 6 | 6.9 | 5.7 | 5.7 | 5.7 | 4.8 | 5.6 | 5.2 | 4.3 | 5.5 | 6.2 | 4.8 |
| 10 | 6.3 | 5.6 | 4.5 | 5.4 | 5.7 | 4.9 | 5.9 | 5.8 | 6 | 6.3 | 5.9 | 5.7 | 5.7 | 4.8 | 5.6 | 5.2 | 4.1 | 5.7 | 6.3 | 4.9 |
| 11 | 6.4 | 5.6 | 4.5 | 5.4 | 5.7 | 4.9 | 5.8 | 5.9 | 6 | 6.3 | 5.7 | 5.7 | 5.8 | 4.8 | 5.5 | 5.2 | 4.1 | 5.6 | 6.3 | 4.8 |
| 12 | 6.5 | 5.6 | 4.5 | 5.6 | 5.7 | 5 | 5.8 | 5.9 | 6 | 6.1 | 5.9 | 5.6 | 5.9 | 4.8 | 5.5 | 5.2 | 4.2 | 5.6 | 6.3 | 4.9 |
| 13 | 6.5 | 5.6 | 4.5 | 5.8 | 5.7 | 5 | 5.8 | 5.8 | 5.9 | 6.8 | 5.6 | 5.6 | 5.9 | 4.8 | 5.5 | 5.3 | 4.1 | 5.6 | 6.3 | 4.9 |
| 14 | 6.6 | 5.7 | 4.5 | 5.5 | 5.8 | 4.9 | 5.8 | 5.8 | 6.1 | 7.5 | 5.6 | 5.7 | 5.8 | 4.8 | 5.6 | 5.5 | 4.2 | 5.8 | 6.3 | 4.9 |
| 15 | 6.6 | 5.5 | 4.5 | 5.5 | 5.8 | 5 | 5.8 | 5.8 | 6.2 | 7.8 | 5.7 | 5.7 | 5.9 | 4.8 | 5.6 | 5.5 | 4.2 | 6 | 6.4 | 4.9 |
| 16 | 6.6 | 5.6 | 4.5 | 5.5 | 5.7 | 5.4 | 5.8 | 5.8 | 6.4 | 8.5 | 5.8 | 5.7 | 5.9 | 4.8 | 5.6 | 5.4 | 4.2 | 6.1 | 6.3 | 4.9 |
| 17 | 6.6 | 5.6 | 4.5 | 6.3 | 5.7 | 5.5 | 5.8 | 5.8 | 6.3 | 9 | 5.8 | 5.7 | 5.7 | 4.8 | 5.6 | 5.4 | 4.2 | 6.2 | 6.3 | 4.9 |
| 18 | 6.6 | 5.6 | 4.5 | 6.8 | 5.7 | 6.1 | 6 | 5.9 | 6.5 | 9.2 | 6.1 | 5.7 | 5.7 | 4.8 | 5.6 | 5.4 | 4.2 | 6.6 | 6.3 | 4.9 |
| 19 | 6.6 | 5.6 | 4.5 | 5.6 | 5.7 | 5.7 | 5.8 | 5.8 | 6.3 | 9.3 | 5.8 | 5.7 | 5.7 | 4.8 | 5.6 | 5.5 | 4.2 | 6.9 | 6.3 | 4.9 |
| 20 | 6.6 | 5.6 | 4.5 | 6.4 | 5.8 | 5.7 | 5.9 | 5.9 | 6.5 | 9.1 | 6.1 | 5.7 | 5.7 | 4.8 | 5.6 | 5.4 | 4.2 | 6.9 | 6.3 | 4.9 |
| 21 | 6.6 | 5.6 | 4.5 | 6.4 | 5.7 | 5.6 | 5.8 | 6 | 6.5 | 9.2 | 5.6 | 5.7 | 5.7 | 4.8 | 5.6 | 5.4 | 4.2 | 6.9 | 6.3 | 4.9 |
| 22 | 6.6 | 5.6 | 4.5 | 6.4 | 5.7 | 5.5 | 5.9 | 6.1 | 6.3 | 9.1 | 5.7 | 5.7 | 5.7 | 4.8 | 5.8 | 5.4 | 4.1 | 7 | 6.3 | 4.9 |
| 23 | 6.6 | 5.6 | 4.5 | 6.5 | 5.7 | 5.2 | 5.8 | 6.7 | 6.2 | 8.7 | 5.6 | 5.7 | 6 | 4.8 | 6.1 | 5.4 | 4.2 | 6.9 | 6.2 | 4.9 |
| 24 | 6.5 | 5.6 | 4.5 | 6 | 5.7 | 5.6 | 5.9 | 6.4 | 6.5 | 8.9 | 5.4 | 5.7 | 6.4 | 4.8 | 6.3 | 5.4 | 4.2 | 7 | 6.2 | 4.9 |
| 25 | 6.4 | 5.6 | 4.5 | 6.1 | 5.7 | 5.6 | 5.9 | 6.6 | 6.5 | 8.8 | 5.3 | 5.7 | 6.6 | 4.8 | 6.1 | 5.4 | 4.1 | 7 | 6.3 | 4.9 |
| 26 | 6.5 | 5.8 | 4.5 | 5.9 | 5.7 | 5.6 | 6.1 | 6.5 | 6.5 | 8.6 | 5.3 | 5.7 | 6.3 | 4.8 | 6.5 | 5.3 | 4.2 | 7.1 | 6.3 | 4.9 |
| 27 | 6.6 | 5.8 | 4.5 | 5.9 | 5.7 | 5.6 | 6.3 | 6.6 | 6.5 | 8.8 | 5.4 | 5.7 |  | 4.8 | 6 | 5.4 | 4.1 | 6.8 | 6.3 | 4.9 |
| 28 | 6.5 | 5.7 | 4.5 | 5.9 | 5.7 | 5.6 | 6.3 | 6.9 | 6.6 | 9 | 5.4 | 5.6 | 7.5 | 4.8 | 6.4 | 5.4 | 4.2 | 7.4 | 6.3 | 4.9 |
| 29 | 6.5 | 5.8 | 4.5 | 6.1 | 5.7 | 6 | 6.6 | 6.8 | 6.9 | 8.7 | 5.3 | 5.7 | 7.7 | 4.8 | 6.6 | 5.3 | 4.2 | 7.2 | 6.3 | 4.8 |
| 30 | 6.5 | 5.7 | 4.5 | 5.9 | 5.7 | 6 | 6.6 | 6.9 | 6.7 | 8.8 | 5.3 | 5.7 | 8.5 | 4.8 | 6.9 | 5.4 | 4.1 | 7.3 | 6.3 | 4.9 |
| 31 | 6.6 | 5.9 | 4.5 | 6.9 | 5.7 | 5.5 | 6.5 | 6.8 | 6.4 | 8.5 | 5.4 | 5.7 | 8.1 | 4.8 | 6.3 | 5.3 | 4.1 | 7.1 | 6.2 | 4.9 |
| 32 | 6.5 | 5.8 | 4.5 | 5.8 | 5.6 | 5.3 | 6.3 | 6.6 | 6.6 | 7.9 | 5.3 | 5.7 | 7.5 | 4.8 | 6.3 | 5.2 | 4.2 | 7.1 | 6.3 | 4.9 |
| 33 | 6.4 | 5.7 | 4.5 | 5.6 | 5.6 | 5.2 | 6.4 | 6.9 | 6.5 | 7.7 | 5.3 | 5.7 | 8.5 | 4.8 | 5.9 | 5.2 | 4.2 | 6.8 | 6.3 | 4.8 |
| 34 | 6.4 | 5.6 | 4.5 | 5.5 | 5.6 | 5.1 | 6.1 | 7.1 | 6.3 | 7.1 | 5.3 | 5.8 | 8.2 | 4.8 | 5.6 | 5.2 | 4.1 | 6.5 | 6.3 | 4.9 |
| 35 | 6.4 | 5.6 | 4.5 | 5.5 | 5.6 | 5.1 | 6 | 6.6 | 6.3 | 6.6 | 5.3 | 5.8 | 7.7 | 4.8 | 5.7 | 5.2 | 4.2 | 6.3 | 6.3 | 4.9 |
| 36 | 6.4 | 5.6 | 4.5 | 5.7 | 5.7 | 5 | 5.9 | 6.7 | 6.4 | 6.4 | 5.3 | 5.7 | 8.7 | 4.8 | 5.6 | 5.2 | 4.1 | 6.1 | 6.3 | 4.8 |
| 37 | 6.4 | 5.6 | 4.5 | 5.6 | 5.7 | 5.1 | 5.8 | 6.2 | 6.2 | 6.4 | 5.3 | 5.7 | 8.5 | 4.8 | 5.8 | 5.2 | 4.1 | 5.9 | 6.3 | 4.8 |
| 38 | 6.4 | 5.5 | 4.5 | 5.5 | 5.7 | 5 | 5.9 | 6.8 | 6.2 | 6.6 | 5.3 | 5.7 | 8 | 4.8 | 5.7 | 5.2 | 4.1 | 5.8 | 6.3 | 4.9 |
| 39 | 6.4 | 5.6 | 4.6 | 5.6 | 5.7 | 5.1 | 5.8 | 6.5 | 6 | 6.5 | 5.3 | 5.7 | 8.2 | 4.8 | 6 | 5.2 | 4.2 | 5.8 | 6.3 | 4.9 |
| 40 | 6.3 | 5.6 | 4.5 | 5.6 | 5.7 | 5.1 | 5.8 | 6.3 | 6 | 6.4 | 5.3 | 5.7 | 7.9 | 4.8 | 5.7 | 5.2 | 4.2 | 5.8 | 6.3 | 4.8 |

*NMES* neuromuscular electrical stimulation, *EE* energy expenditure
