# Supplementary material for: Metabolic response during neuromuscular electrical stimulation in critically ill patients with deep sedation: a prospective observational study
Source: Intensive Care Med Exp. 2026 Nov 10;14:145. doi: 10.1186/s40635-026-01000-5 (PMC13748173; doi:10.1186/s40635-026-01000-5)
Supplement: Supplementary file 4 [file 40635_2026_1000_MOESM4_ESM.docx]

**Additional File 4.** The change in hemodynamic and metabolic parameters associated with NMES in an individual session.

| No | Energy Expenditure (kcal/day) | VO_2_ (ml/min) | HR　(bpm) | Percentage increase in HR (%) | SAP (mmHg) | Percentage increase in SAP (%) | MAP (mmHg) | Percentage increase in MAP (%) | EtCO_2_ (mmHg) | Percentage increase in EtCO_2_ (%) | MV (L/min) | Percentage increase in MV (%) |
| --- | --- | --- | --- | --- | --- | --- | --- | --- | --- | --- | --- | --- |
| 1 | 212.1 | 32.1 | 0.2 | 0.3 | −8.8 | −7.3 | −8.0 | −9.9 | 4.2 | 13.5 | 0.08 | 1.2 |
| 2 | 148.1 | 25.4 | 1.3 | 1.8 | 9.6 | 9.3 | 3.1 | 4.8 | −0.5 | −1.4 | 0.05 | 0.9 |
| 3 | 109.4 | 17.9 | 0.6 | 1.0 | 4.8 | 4.3 | 3.7 | 6.2 | 2.6 | 6.7 | −0.03 | −0.6 |
| 4 | 259.7 | 38.0 | 1.6 | 2.4 | −3.3 | −3.5 | −3.8 | −5.7 | 3.7 | 9.4 | 0.49 | 8.8 |
| 5 | 156.9 | 27.1 | −1.8 | −2.3 | −1.5 | −1.4 | −1.4 | −2.2 | 2.1 | 5.6 | 0.02 | 0.3 |
| 6 | −178.7 | −28.5 | 0.7 | 0.6 | −4.4 | −3.7 | −3.0 | −4.1 | 0.1 | 0.2 | 0.58 | 11.7 |
| 7 | 143.1 | 22.4 | 7.9 | 8.8 | −8.3 | −6.1 | −6.8 | −7.0 | 2.1 | 5.9 | 0.12 | 2.0 |
| 8 | 61.6 | 15.5 | 2.7 | 2.9 | 0.3 | 0.2 | −0.3 | −0.4 | 1.7 | 4.9 | 0.36 | 6.1 |
| 9 | 251.9 | 41.1 | 5.6 | 7.2 | 8.1 | 7.9 | 2.2 | 3.3 | 7.5 | 19.6 | 0.37 | 6.2 |
| 10 | 544.8 | 80.6 | 5.2 | 6.2 | 4.7 | 4.9 | 3.9 | 6.5 | 0.9 | 2.2 | 1.96 | 30.4 |
| 11 | 238.8 | 37.7 | 1.3 | 1.8 | 7.9 | 6.8 | 7.9 | 10.5 | −5.9 | −12.9 | −0.12 | −2.1 |
| 12 | 200.8 | 29.3 | 0.9 | 1.2 | −3.2 | −2.3 | −1.5 | −2.1 | 4.9 | 12.1 | −0.02 | −0.3 |
| 13 | 314.3 | 45.0 | 2.3 | 3.6 | −17.4 | −10.9 | −8.1 | −9.8 | 4.6 | 12.4 | 0.52 | 9.1 |
| 14 | 124.3 | 20.1 | 1.8 | 2.6 | −4.7 | −3.8 | −4.6 | −5.9 | 3.3 | 8.0 | −0.02 | −0.4 |
| 15 | 394.3 | 59.1 | 3.4 | 3.6 | 5.7 | 4.5 | 2.6 | 2.8 | 9.1 | 25.2 | 0.31 | 5.5 |
| 16 | 177.6 | 27.4 | −0.8 | −1.5 | −9.5 | −6.6 | −8.4 | −10.1 | 2.4 | 6.4 | 0.13 | 2.5 |
| 17 | 248.0 | 32.7 | 1.6 | 2.4 | −20.3 | −18.4 | −14.1 | −17.8 | 6.1 | 15.1 | 0.03 | 0.7 |
| 18 | 208.0 | 29.0 | −2.7 | −3.9 | −24.1 | −16.4 | −14.6 | −16.1 | 1.5 | 4.0 | 0.78 | 13.3 |
| 19 | 229.2 | 36.1 | 0.3 | 0.5 | −17.3 | −13.4 | −11.4 | −14.0 | 4.5 | 13.9 | −0.04 | −0.6 |
| 20 | 220.7 | 35.9 | 0.1 | 0.2 | −13.9 | −12.9 | −12.4 | −17.8 | 5.1 | 15.7 | 0.03 | 0.6 |
| 21 | 46.1 | 11.0 | −0.4 | −0.6 | −3.5 | −2.5 | −8.0 | −8.2 | 3.0 | 10.2 | −0.05 | −0.7 |
| 22 | 153.1 | 26.6 | 2.5 | 2.5 | −3.4 | −2.8 | −2.9 | −4.1 | 2.0 | 5.9 | 0.07 | 1.1 |
| 23 | 355.4 | 55.8 | −1.1 | −1.6 | −10.5 | −10.1 | −8.2 | −12.6 | 6.8 | 21.6 | −0.15 | −1.9 |
| 24 | 185.7 | 29.5 | 0.8 | 1.1 | −7.6 | −6.4 | −8.6 | −10.6 | 4.0 | 9.0 | 0.006 | 0.1 |
| 25 | 296.7 | 47.1 | 12.4 | 20.8 | −3.6 | −3.6 | −0.2 | −0.3 | 7.4 | 21.1 | −0.12 | −2.3 |
| 26 | 456.1 | 70.5 | 4.5 | 7.7 | −9.9 | −7.0 | −8.5 | −10.8 | 9.5 | 24.7 | 0.34 | 6.7 |

Energy expenditure, VO_2_, HR, SAP, MAP, EtCO_2_, and MV are defined as the change in each parameter associated with NMES. *bpm* beats per minute, *EtCO_2_* end tidal carbon dioxide, *HR* heart rate, *MAP* mean arterial pressure, *MV* minute volume, *NMES* neuromuscular electrical stimulation, *SAP* systolic arterial pressure, *VO_2_* oxygen consumption.
